# Assessing the comparative effectiveness of Tai Chi versus physical therapy for knee osteoarthritis: design and rationale for a randomized trial

**DOI:** 10.1186/1472-6882-14-333

**Published:** 2014-09-08

**Authors:** Chenchen Wang, Maura D Iversen, Timothy McAlindon, William F Harvey, John B Wong, Roger A Fielding, Jeffrey B Driban, Lori Lyn Price, Ramel Rones, Tressa Gamache, Christopher H Schmid

**Affiliations:** Center for Integrative Medicine and Division of Rheumatology, Tufts Medical Center, Tufts University School of Medicine, Boston, MA USA; Department of Physical Therapy, Movement and Rehabilitation Sciences, Northeastern University, Boston, MA USA; Division of Rheumatology, Immunology & Allergy, Brigham & Women’s Hospital, Harvard Medical School, Boston, MA USA; Division of Clinical Decision Making, Department of Medicine, Tufts Medical Center, Institute for Clinical Research and Health Policy Studies, Tufts Clinical and Translational Science Institute, Tufts University, Boston, Medford, MA USA; Nutrition, Exercise Physiology, and Sarcopenia Laboratory, Jean Mayer USDA Human Nutrition Research Center on Aging, Tufts University, Boston, USA; The Institute for Clinical Research and Health Policy Studies, Tufts Clinical and Translational Science Institute Tufts Medical Center, Tufts University, Boston, MA USA; Center for Mind–Body Therapies, Providence, RI USA; Department of Biostatistics and Center for Evidence Based Medicine, Brown University School of Public Health, Providence, RI USA

**Keywords:** Tai Chi, Physical therapy, Knee osteoarthritis, Comparative effectiveness research, Randomized controlled trial

## Abstract

**Background:**

Knee osteoarthritis (OA) causes pain and long-term disability with annual healthcare costs exceeding $185 billion in the United States. Few medical remedies effectively influence the course of the disease. Finding effective treatments to maintain function and quality of life in patients with knee OA is one of the national priorities identified by the Institute of Medicine. We are currently conducting the first comparative effectiveness and cost-effectiveness randomized trial of Tai Chi versus a physical-therapy regimen in a sample of patients with symptomatic and radiographically confirmed knee OA. This article describes the design and conduct of this trial.

**Methods/Design:**

A single-center, 52-week, comparative effectiveness randomized controlled trial of Tai Chi versus a standardized physical-therapy regimen is being conducted at an urban tertiary medical center in Boston, Massachusetts. The study population consists of adults ≥ 40 years of age with symptomatic and radiographic knee OA (American College of Rheumatology criteria). Participants are randomly allocated to either 12 weeks of Tai Chi (2x/week) or Physical Therapy (2x/week for 6 weeks, followed by 6 weeks of rigorously monitored home exercise). The primary outcome measure is pain (Western Ontario and McMaster Universities WOMAC) subscale at 12 weeks. Secondary outcomes include WOMAC stkiffness and function domain scores, lower extremity strength and power, functional balance, physical performance tests, psychological and psychosocial functioning, durability effects, health related quality of life, and healthcare utilization at 12, 24 and 52 weeks.

**Discussion:**

This study will be the first randomized comparative-effectiveness and cost-effectiveness trial of Tai Chi versus Physical Therapy in a large symptomatic knee OA population with long-term follow up. We present here a robust and well-designed randomized comparative-effectiveness trial that also explores multiple outcomes to elucidate the potential mechanisms of mind-body effect for a major disabling disease with substantial health burdens and economic costs. Results of this study are expected to have important public health implications for the large and growing population with knee OA.

**Trial registration:**

ClinicalTrials.gov identifier: NCT01258985

**Electronic supplementary material:**

The online version of this article (doi:10.1186/1472-6882-14-333) contains supplementary material, which is available to authorized users.

## Background

Knee osteoarthritis (OA) is a major age-related public health problem resulting in pain, functional limitations, disability, and decreased quality of life. An estimated 27 million people in the United States (US) have OA [1], and nearly half of all Americans are projected to develop knee OA during their lifetime [2]. Based on the US Medical Expenditure Panel Survey, associated insurer and out of pocket healthcare costs account for more than $185 billion per year with another $10 billion lost from absenteeism at work [3, 4].

Currently available medical treatments have limited influence on the trajectory of the disease. Nonsteroidal anti-inflammatory drugs and acetaminophen often fail to relieve symptoms and may lead to serious adverse effects [5–7]. Physical therapy, a prominent component of knee OA guidelines, includes aerobics and muscle strengthening exercises. Although clinical trials involving Physical Therapy have been performed with heterogeneity in frequency, intensity, and duration of treatment, current evidence suggests that Physical Therapy produces only moderate benefits for pain and physical functioning with limited durability of effects [8–10].

Therefore, identifying new and effective treatments, particularly non-pharmacologic treatments, to further improve and maintain function and quality of life in people with knee OA remains a national priority, as evidenced by its placement on the Institute of Medicine’s list of 100 national priorities for comparative effectiveness research.

Our previous work indicates that Tai Chi, a form of mind-body exercise that combines deep diaphragmatic breathing and relaxation with slow, gentle, graceful movements, can improve both physical and psychological health among patients with chronic conditions [11]. Significant improvements have been reported in balance, strength, cardiovascular and respiratory functioning, flexibility, pain, depression, anxiety and arthritic symptoms [12–20].

In our preliminary randomized trial, participants with knee OA who completed Tai Chi exhibited greater improvements in pain, physical function, depression, self-efficacy, and health status compared with an attention control group at 12 weeks. Primary and secondary outcomes also showed durable benefits at 24 and 48 weeks [18]. Furthermore, our recent meta-analysis of six Tai Chi studies including 382 participants demonstrated large and significant pain reductions [Effect Size = 0.72 (95% CI 0.97, 0.47)] after 8 to 12 weeks of Tai Chi training compared with a variety of controls with a heterogeneity score (I^2^) of 0% [21].

These previous trials demonstrate that our adaptation of Tai Chi has the potential to become a unique, logistically feasible way of providing standardized exercises with a complementary mind-body approach for the management of knee OA. The physical component provides exercise that is consistent with recommendations for knee OA (flexibility, muscle conditioning, balance, and aerobic cardiovascular exercise) [22], while the mind component has the potential to increase psychological well-being, life satisfaction, and perceptions of health [14, 23].

Therefore, we are currently conducting the first comparative effectiveness and cost-effectiveness trial in a large symptomatic knee OA population. We hypothesize that Tai Chi will outperform Physical Therapy as typically practiced in a clinical setting. We aim to demonstrate that, compared with Physical Therapy, Tai Chi may be an effective and cost-effective therapy for managing pain and reducing the functional limitations that impact the quality of life for millions of individuals with knee OA.

In this paper we present the design and detailed protocol of the first comparative effectiveness and economic analysis of a randomized controlled trial of Tai Chi versus a Physical Therapy regimen in adults with knee OA. It is expected that the study will fill important knowledge gaps and generate critical insights to inform clinical decision making for knee OA. The results from this trial will be reported at the completion of the study in accordance with the Consolidation of Standards for Reporting Trials guideline [24].

## Methods/Design

### Study design overview

This study is a single-center, 52-week, randomized controlled trial. Over four years, 180 older adults with symptomatic knee OA are randomly assigned to Tai Chi (2x/week for 12 weeks) or a physical-therapy regimen in a clinical setting (2x/week for 6 weeks followed by 6 weeks of home exercise).

The primary outcome is pain measured by the well-validated Western Ontario and McMaster University Index (WOMAC) pain subscale at 12 weeks [25]; secondary outcomes include WOMAC stiffness and function domain scores, lower extremity strength and power, physical performance tests, psychosocial functioning (mental health, self-efficacy, stress, depression, mindfulness, social support), durability of effect after treatment ends, healthcare utilization, outcome expectation, adherence, and occurrence of adverse events. Covariates include age, gender, body mass index, and comorbidities. We are also examining the economic impact of Tai Chi versus a Physical Therapy regimen based on healthcare utilization data for the treatment of knee OA. Outcome measurements are collected at baseline, every week during the intervention period (WOMAC), upon completion of the 12-week program, and at 24 and 52 weeks. The staff conducting the physical function assessments and the statistician are blinded to treatment assignment. The specific endpoints and their conceptualization as outcomes or intermediaries are presented in Table 1.

**Table 1:** **Sequence of trial measurements for primary and secondary outcomes** ^**1**^

	Baseline	Intervention	Week 12	Week 24	Week 52
*Time (Months)*	*-1*	*1-3*	*3*	*6*	*12*
**Primary outcome variable**					
WOMAC – Pain^2^	x	x	x	x	x
**Secondary outcome variables**					
WOMAC – Physical Function	x	x	x	x	x
WOMAC – Stiffness	x	x	x	x	x
Patients’ Global OA Severity	x		x	x	x
SF-36	x		x	x	x
Beck II Depression Inventory	x		x	x	x
Perceived Stress Scale	x		x	x	x
Chronic Pain Self-Efficacy	x		x	x	x
Social Support	x		x	x	x
OES for Exercise	x		x	x	x
OES for Tai Chi/PT		x			
PROMIS^3^	x		x	x	x
Health Assessment Questionnaire	x		x	x	x
NEO Five-Factor Inventory^4^	x		x	x	x
Five Facet Mindfulness	x		x	x	x
Credibility-Expectancy^5^		x	x		
Pre-Clinical Disability	x		x	x	x
Self-Reported Alignment	x				
CHAMPS	x		x	x	x
Functional Performance Tests^6^	x		x	x	x
Body Mass Index	x		x	x	x
Medications	x		x	x	x
Adverse Events	x	x			
Adherence	x	x	x	x	x

^1^WOMAC = Western Ontario and McMaster Universities Osteoarthritis Index, SF-36 = Medical Outcome Survey Short-Form 36, PROMIS = Participant-Reported Outcomes Measurement Information System, OES = Outcome Expectations Scale, PT = Physical Therapy.

^2^WOMAC Pain is the primary outcome at 12 weeks; the other collection times are secondary outcome variables.

^3^PROMIS questionnaires include PROMIS Pain Impact, PROMIS Physical Function, PROMIS Depression, PROMIS Anxiety, PROMIS Sleep Disturbance, Satisfaction with Social Roles, and PROMIS HAQ.

^4^The NEO Five-Factor Inventory and Self-Reported Alignment are given out once over the course of the study at the earliest available evaluation period.

^5^The Credibility-Expectance Questionnaire are given out before the start of the first intervention session.

^6^Functional Performance Tests include timed chair stand, berg balance, six minute walk, 20-meter walk, gait analysis, muscle strength and power, and postural sway.

The study setting is an urban tertiary care academic hospital, Tufts Medical Center, in Boston Massachusetts. The study received ethics approval from the Tufts Medical Center/Tufts University Institutional Review Board.

### Study sample

Individuals aged ≥ 40 years who meet American College of Rheumatology criteria for symptomatic knee OA (pain on more than half the days of the past month during at least one of the following activities: walking, going up or down stairs, standing upright, or lying in bed at night) [26] and who have radiographic evidence of tibiofemoral or patellofemoral OA (defined as the presence of a definite osteophyte in the tibiofemoral compartment and/or the patellofemoral compartment, as assessed on standing anterior/posterior and lateral or sunrise views) are invited to participate [26]. The study rheumatologist (WH), who conducts the clinical examinations, confirms the presence of knee pain or discomfort and that the individual would be physically able to participate in Tai Chi and Physical Therapy programs. All participants are required to have a WOMAC pain subscale score ≥ 40 (visual analog version) on at least one of five questions (range 0 to 100 each, higher indicating more pain) at the baseline evaluation in order to participate.

Individuals are excluded if they have: 1) prior experience with Tai Chi or other similar types of complementary and alternative medicine such as Qi Gong or yoga, or prior experience with Physical Therapy programs for knee OA in the past year; 2) serious medical conditions that limit his/her ability to safely participate in either the Tai Chi or Physical Therapy programs, including dementia, neurological disease, symptomatic heart or vascular disease (angina, peripheral vascular disease, congestive heart failure), severe hypertension, recent stroke, severe insulin-dependent diabetes mellitus, psychiatric disease, renal disease, liver disease, active cancer or anemia; 3) any intra-articular steroid injections or reconstructive surgery in the three months prior to baseline screening on the most severely affected knee (study knee); 4) any intra-articular hyaluronic acid injections in the six months prior to baseline screening; 5) inability to pass the Mini-Mental Status examination (with a score below 24) [27]; or 6) inability to walk without a cane or other assistive device for the entire duration of the baseline assessments; 7) not English-speaking; 8) positive pregnancy test or planning pregnancy; 9) enrollment in any other clinical trial within the last 20 days; 10) plan to permanently relocate from the region during the trial period.

#### Radiographs

Anterior-posterior (AP) and lateral standing and sunrise view knee radiographs are obtained at the initial screening examination (the baseline assessment) using the Framingham study protocol [28] and are screened by the study rheumatologist (WH) for presence of a definite osteophyte [29, 30]. Substitution of another clinical knee radiograph previously obtained is permitted as long as a definite osteophyte can be readily identified by the study physician, even if performed using a different radiographic protocol.

The weight-bearing radiographs are also scored using the Kellgren and Lawrence (K/L) grading system for global tibiofemoral radiographic severity. The K/L score is determined for each knee compartment based on osteophyte formation, joint space width, and subchondral bone scleroses [31]. All scores reported are for the most severely affected knee (study knee). Radiographs are also scored according to the OARSI atlas [32] for osteophyte size and joint space narrowing in the tibiofemoral and patellofemoral compartments.

#### Knee examination

Knee examinations are performed at baseline visits. Prior to treatment assignment, the study rheumatologist (WH) assesses the presence and severity of the knee joint abnormalities relevant to knee OA and safe participation in the study, including ligamentous laxity, meniscal abnormalities, flexion contractures, alignment abnormalities and surgical scars.

### Recruitment strategies

Combinations of advertising strategies that have been found to be successful in prior recruitment initiatives for knee OA clinical trials are employed. These strategies include flyers within the hospital and advertisements in the print and online media. To ensure adequate enrollment of a diverse study population, including underrepresented groups, advertisements are placed in a wide range of media outlets including: myHospitalWebsite, Craigslist, Facebook, Tufts Medical Center website, SAMPAN (a Chinese newspaper), the Boston Metro and Boston Fifty Plus newspapers and a booth at a senior expo. Participants are also recruited from the rheumatology clinic patient database at Tufts Medical Center. To accomplish this, we obtain a Health Insurance Portability and Accountability Act waiver to flag the charts of patients with a billing code of OA who have attended the Rheumatology Clinic within the last year, and approach them for participation. Any interested respondents receive information about the study and answer a brief, scripted survey to determine their eligibility for the trial (Additional file 1). This screening survey includes items whose predictive values for knee OA are known from population-based data [33].

### Enrollment and the informed consent process

Participants are enrolled in groups of 20 or more participants per cycle in order to ensure a full classroom setting for the group intervention and a one to one ratio for the two interventions. In the three-week period prior to the start of the intervention period, baseline assessments for a group of 30 to 40 pre-screened participants are performed in order to obtain an eligible cohort of participants to randomize. Prior to any information being collected, the principal investigator (CW) or study coordinator completes the informed consent process. Each person who agrees to participate provides informed consent. After providing informed consent, the principal investigator or study staff screen participants to confirm that potential participants meet the eligibility criteria listed above.

After verifying the participant’s potential eligibility, radiographs are obtained as described above for confirmation of radiographic OA. If a participant has a definite osteophyte on any view in the symptomatic knee, the participant is randomized through the process described below.

### Randomization

Randomizing participants for a group intervention (Tai Chi) presents some challenges, as does having an active comparator that is not a group intervention (Physical Therapy). Participants who pass eligibility criteria including the radiographic and physical examination screen are offered enrollment in the study. Randomization occurs after the baseline evaluation.

The study statistician (CS) randomizes assignments using a sequence randomly generated in the R statistical package [34]. These assignments are then sent to a study staff member other than the study coordinator or PI, who puts them into sealed, opaque envelopes with date and signature labels placed over the seals of the envelopes. The study coordinator then contacts eligible participants by phone, confirms that each one still wishes to participate in the study, opens the consecutive randomization envelope, and informs the individuals of their group assignment. The study coordinator also informs the participants of the schedule for their training sessions (including date and time). These procedures are precisely described in a Manual of Operations.

The study is conducted in nine cycles of at least 20 participants each for a 12-week treatment course. Participants from this pool are randomized in a 1:1 ratio to Tai Chi or Physical Therapy. One of the unanswered questions from our previous studies was whether the observed benefit of Tai Chi depended on the instructor or whether it could be generalized. Systematic differences across instructors would imply that the amount of treatment benefit might depend on the skill level of the instructor; lack of differences would imply that the benefit might apply more generally. Over the course of the entire study, each Tai Chi group is randomly assigned to one of the three Tai Chi instructors so that each instructor teaches one group of approximately 10 participants every three cycles. To evaluate between-therapist differences in Physical Therapy, participants randomized to that intervention are further randomized to receive treatment by one of three physical therapists (two at the hospital Physical Therapy Clinic and one at a community therapy clinic).

### Study intervention

Both Tai Chi and Physical Therapy interventions run concurrently to avoid seasonal influences on disease severity. During the first six weeks, both treatment arms receive supervised exercise (either Tai Chi in groups or an individual Physical Therapy regimen with the recommendation to practice these exercises daily for 20 minutes at home). After six weeks, participants randomized to Physical Therapy transition to a personalized home exercise program while the Tai Chi group continues in the group format. Participants in the Physical Therapy group are instructed to engage in home exercises four times a week for 30 minutes and are contacted weekly by phone during their six-week home exercise period to ascertain adherence. Adherence data included frequency, duration of exercise and types of exercises performed. The six week course of Physical Therapy followed by home therapy is consistent with the type and duration of intervention typically received in a normal clinical setting, which is often, in the United States, limited by insurance or other payers to 6 weeks.

Participants are able to continue routine medications such as nonsteroidal anti-inflammatory drugs (NSAIDS) and acetaminophen, and maintain their usual treatment visits with their primary care physician or rheumatologist throughout the study. Participants are not required to wash out of their pain medications prior to formal assessment visits. The research staff records any changes made to treatment but do not change or recommend changes in medical therapy.

#### Tai Chi intervention

The participants randomized to Tai Chi practice at Tufts Medical Center. A detailed description of a standardized Tai Chi protocol was prepared and well-tested in our previous trials [12, 16, 18, 35]. The intervention classes take place at varied one hour blocks during afternoon business hours, Monday-Friday.

The three Tai Chi instructors each have extensive experience (>20 years) conducting Tai Chi mind-body programs and follow a Tai Chi protocol specifically designed for individuals with knee OA. In addition, all three instructors completed the required research and human subject protection training prior to initiation of the intervention classes.

To ensure that instructors are prepared to teach a robust, standardized Yang-style Tai Chi treatment program for patients with knee OA, we conduct a training session with the three Tai Chi instructors to thoroughly review concepts of knee OA and a standardized teaching menu at the beginning of the study and reviewed as needed throughout the course of the study. All sessions are monitored by regularly reviewing video recordings and providing feedback throughout the study to ensure proper instruction.

Each Tai Chi session lasts 60 minutes and continues twice a week for 12 weeks. Participants are also provided with printed materials on the Tai Chi Mind-Body program, including Tai Chi principles, practicing techniques, and safety precautions for participants with knee OA. In the first session, the Tai Chi instructor explains exercise theory and Tai Chi procedures. For the remaining sessions, the procedures include the following components: (1) warm-up and a review of Tai Chi principles and techniques, (2) Tai Chi movement, (3) breathing techniques, and (4) various relaxation methods. All program components are derived from classical Yang style Tai Chi 108 postures [36]. Because of time limitations in executing a study on the use of Tai Chi and based on the literature [11] we condensed the 108 postures of Classical Yang style Tai Chi to 10 forms that could be learned by elderly subjects with knee osteoarthritis within 12 weeks. The 10 forms were selected because (1) they are easily comprehensible; (2) clearly represent progressive degrees of stress to postural stability, with weight bearing moving from bilateral to unilateral supports; and (3) seem to emphasize increasing magnitude of truck and arm rotation with diminishing base of support and, as such, will potentially improve physical function without excessively stressing the joints. An outline of the Tai Chi exercise program is shown in Additional file 2.

All participants are encouraged to maintain their usual physical activities and to perform no new additional strength training other than their Tai Chi exercises. Participants are also instructed to practice Tai Chi for at least 20 minutes per day at home. Tai Chi instructors remind subjects in class of daily practice and give out weekly assignments of Tai Chi poses to practice. The data collected for class attendance are recorded and verified using standard case report forms that include a participant sign in sheet as well as a staff completed attendance sheet to confirm accurate attendance recordings.

After completing the 12 week intervention (24 treatment sessions), participants are asked to continue with their exercises without direct supervision. The research team monitors these participants once a month with home calls until the 52-week follow-up evaluation, which is described in further detail below.

#### Physical Therapy regimen

Participants randomized to the Physical Therapy regimen receive a musculoskeletal examination consistent with contemporary Physical Therapy practice [9]. The Physical Therapy intervention takes place at either the hospital or a specific community therapy clinic.

The three Physical Therapists are experienced in outpatient orthopedic Physical Therapy, each possessing more than 10 years of clinical outpatient experience. Additionally, all three Physical Therapists completed the required research and human subject protection training prior to the initiation of the intervention sessions.

Prior to recruitment, the supervising Physical Therapy research scientist (MDI) developed an evaluation form (and corresponding coding protocol) based on contemporary orthopedic Physical Therapy practice using the Tufts Medical Center evaluation form as a template (provided in Additional file 3). A series of training sessions was conducted with the three study Physical Therapists to discuss the content of the examination form, standardize documentation procedures and develop a consensus for performing special tests. Specifically, the Physical Therapy team came to a consensus on a single method for each of the special tests used in a standard Physical Therapy examination for muscle flexibility, ligamentous and meniscal integrity and posture. The initial examination is performed by one of the three study Physical Therapists and generally lasts one hour. The supervising therapist reviews the examination forms for the first 3–4 subjects enrolled per Physical Therapist to check for consistency in recording data and to refine the coding protocol. During each recruitment cycle, the supervising Physical Therapy research scientist visits each therapist to ensure documentation of examination procedures is consistent with the established protocol.

Depending on the diagnostic findings in the initial exam, the Physical Therapist develops tailored personalized Physical Therapy regimens to address specific treatment goals developed collaboratively with the participant and consistent with current recommended guidelines for knee OA [8].

The remainder of the Physical Therapy intervention consists of 30-minute sessions, held twice a week for six weeks at the designated Physical Therapy clinic. The multi-modal Physical Therapy regimen is designed to produce the desired outcomes and in general consists of joint mobilizations, active and passive knee and hip range-of-motion exercises, hip and knee strengthening exercises, stretching lower limb functional exercises, stationary biking, balance exercises, and neuromuscular training. All of the activities are designed to be functional and mutually reinforcing, with repeated and sustained gentle challenges to the end-range of movement. The Physical Therapist increases the repetitions and intensity of strengthening exercises on the basis of participant tolerance. For example, open chain exercises (e.g., straight leg raises) are progressed to closed chain exercises (e.g., mini-wall squats) that are further progressed to activities such as mini-squats. Balance activities are progressed from wide stance to narrow stance (or tandem stance) and eventually to single leg balance (with minor support to no support). The brief examples of the flexibility and straight plane strengthening exercises are shown in Additional file 4.

At each session, the treating Physical Therapist examines the participant for adverse signs and symptoms, such as increased pain, joint effusion, and increased skin temperature over the knee joints. These signs and symptoms of knee OA need to be stable or decreasing before manual therapy or exercise is progressed. The supervising Physical Therapist closely monitors the knee exercise program during each recruitment cycle, visiting each therapist once a cycle to observe the intervention sessions.

All participants are encouraged to maintain their usual physical activities and to perform no new additional strength training other than their Physical Therapy exercise. Participants are encouraged to practice these exercises daily for 20 minutes at home. The Physical Therapists remind participants during outpatient sessions of daily practice. The data collected for session attendance are recorded and verified using standard case report forms completed by the Physical Therapist on a weekly basis.

After six weeks, the Physical Therapy regimen transitions to a six-week home exercise program. Participants receive illustrations of exercises and are instructed to exercise four times per week for 30 minutes. Thus, the home program (4 times per week for 30 minutes over 6 weeks plus the initial 6-week, individual Physical Therapy) is equivalent in dose to the Tai Chi intervention (twice a week for 60 minutes over 12 weeks). The home exercise program is essentially the same as the outpatient program with guidelines for progressing exercises provided by the Physical Therapists. The individualized home exercise programs are monitored weekly by study staff through phone interview using standardized forms including questions about frequency, exercises completed, and adverse events. At the end of the six-week home program, participants are asked to integrate their exercise into their daily routine to enable them to continue with their exercises throughout the follow up period. The research team monitors these participants once a month with home calls until the 52-week follow-up evaluation.

### Measurements

Knee OA outcome measurements are drawn from the core set recommended by the Osteoarthritis Research Society International [25], and focus on pain, physical function, and patients’ overall assessment of their knee OA severity. Every participant is evaluated at baseline (prior to starting either intervention), after completing the intervention (12 weeks later), and at a 24 week and 52 week follow-ups (Table 1).

### Primary outcome

The primary outcome measure is change in the WOMAC pain subscale between baseline and 12 weeks. The WOMAC (version VA3.1) is a validated, self-administered, visual analogue scale specifically designed to evaluate knee and hip OA [37, 38]. It has three subscales that are analyzed separately: pain (score range, 0–500), stiffness (0–200), and function (0–1700), with higher scores indicating more severe disease. In addition, WOMAC pain is also assessed weekly during the intervention period for both groups of participants as well as at the 24 and 52 week time points as secondary outcomes.

### Secondary outcomes

The secondary outcome measures include psychological, psychosocial, muscle strength, functional balance, and neuromuscular variables as well as the healthcare utilization of the treatment for knee OA. They are described in the following sections.

#### Psychological and psychosocial functioning measures

The *Patient’s Global Assessment* is a visual analogue scale that measures the level of Knee OA severity on a 10 point scale with 10 reflecting the most extreme severity and 0 reflecting no severity.

*Health Related Quality of Life (HRQL)* assessments are made using the Medical Outcome Study Short Form 36 Short-Form Health Survey (SF-36) [39]. The SF-36 is a self-administered, 36-item questionnaire that assesses the concepts of physical functioning, role limitations due to physical problems, social function, bodily pain, general mental health, role limitations due to emotional problems, vitality, and general health perceptions. Note that both the physical and mental component summaries can be combined. Scores range from 0 to 100, with higher scores indicating better health status [40].

The *Beck II Depression Inventory* is a 21-question, validated, self-report instrument that measures the severity of depressive symptoms. Higher scores reflect a greater degree of symptom severity [41].

The *Perceived Stress Scale (PSS)* is the most widely used psychological instrument for measuring the perception of stress. The scale also includes a number of direct queries about current levels of experienced stress. For this instrument, higher scores reflect a greater degree of symptom severity. The PSS shows high levels of internal consistency (alpha = 0.92) [42].

The *Chronic Pain Self-Efficacy Scale (CPSS)* is a modified version of The Arthritis Self-Efficacy Scale that has been validated for patients with chronic pain [43]. It contains 8 questions divided into 3 subscales (pain coping, physical functioning, and coping with symptoms). The score is obtained by means of a Likert scale with a range of 0–10, where higher scores indicate better self-efficacy.

*The Medical Outcome Study Social Support Survey* assesses social support by using the Social Support for Physical Activity Scale adapted from Sherbourne and colleagues [44]. It comprises 19 questions rated from 0 to 5 to assess the influences of family and friends have on patients as they performed regular physical activity. Higher scores reflect more perceived social support from these individuals.

*Outcome expectations* are beliefs that carrying out a specific behavior such as physical activity will lead to a desired outcome. The brief, validated, outcome expectations scale [45] contains 9 questions that ask about physical and mental benefits and are used to assess outcome expectations. Scores can range from 1 to 5, with 1 indicating low outcome expectations for the exercise and 5 suggesting high outcome expectations. This questionnaire is used prior to randomization to assess the outcome expectation for any exercise intervention. It is also assessed after randomization prior to the first session to assess the outcome expectation for the assigned intervention.

Participants enrolled in the trial also complete six *Participant-Reported Outcomes Measurement Information System (PROMIS)* static short-forms, version 1.0 instruments including PROMIS Pain Impact, Physical Functioning, Emotional Distress-Anxiety, Emotional Distress-Depression, Sleep Disturbance, Satisfaction with Participation in Social Roles, and. All of the included PROMIS instruments contain 5-point Likert-based items capturing intensity, frequency, or duration. The instruments use a seven-day recall period, with the exception of PROMIS Physical Function, which does not reference any timeframe. Higher scores reflect greater symptom severity across the pain, anxiety, depression and sleep disturbance scales. Higher scores reflect better outcomes for the satisfaction and physical function scales.

The *Health Assessment Questionnaire (HAQ),* developed originally at Stanford in the late 1970’s to assess patients with rheumatoid arthritis, has been validated in a broad range of rheumatic and non-rheumatic disease populations [46, 47]. In particular, the HAQ Disability Index (HAQ-DI) assesses disability and the full HAQ collects data on disability, pain, medication effects, mortality, and healthcare resource use (care costs), [46] including both direct (e.g. physician visits, medication use, arthroplasty) and indirect costs (e.g. loss of productivity) and mortality. We substitute the Improved HAQ for the original HAQ-DI to assess disability because of its improved responsiveness and precision [48–50]. The *NEO Five-Factor Inventory* is a validated 60-item questionnaire that measures the five domains of personality including Neuroticism, Agreeableness, Conscientiousness, Extraversion, and Openness [51]. It consists of five 12-item, 5-point Likert scales that measure each of the domains.

The *Five Facet Mindfulness Questionnaire (FFMQ)* is a validated, 39-item questionnaire that measures five facets of mindfulness: observe, describe, act aware, nonjudge, and nonreact [52]. Participants answer each of the questions on a five-point Likert scale with higher scores reflecting higher mindfulness.

The *Credibility/Expectancy Questionnaire (CEQ)* is a validated, 6-item instrument that assesses how believable, convincing, and logical the treatment seems to the participant as well as what improvements the participant thinks will be achieved. This questionnaire has been adapted to reflect the participant population of this study. Higher scores reflect greater credibility and expectancy by the participant [53].

The *Pre-Clinical Disability (PCD) Questionnaire* is an adapted 12-item, yes/no questionnaire that assesses whether participants have changed the way or how often they do a series of daily activities such as climb a flight of stairs or carry groceries [54]. More positive answers reflect greater preclinical disability.

The *Self-Reported Alignment Questionnaire* is an 8-item questionnaire in which participants identify the angle of their knees and feet with those shown in the pictures for their current adult life as well as their early adult life [55].

The *CHAMPS Physical Activity Questionnaire for Older Adults (CHAMPS)* is a validated, 40-item questionnaire that measures weekly physical activity levels for older adults by calculating caloric expenditure [56] and frequency of various common exercises completed by older adults such as swimming or walking. Higher scores reflect greater physical activity levels.

#### Physical performance

Physical performance assessments include the timed chair stand, the six-minute walk test, 20-meter walk test, functional balance, and lower extremity strength and power.

The *timed chair stand tests* measures time taken to complete ten full stands from a sitting position and is a reliable measure of lower body strength and dynamic balance [57, 58]. The recorded time is the average on two tries.

*The six-minute walk test* is a reliable measure of functional exercise capacity [59, 60]. Participants are asked to walk as fast and as far as possible within the six-minute period. Participants are given verbal encouragement every minute throughout the 6 minutes and are informed of the remaining time every minute. The distance covered at the end is noted and recorded.

*The 20-meter walk test* is a reliable measure of gait speed. Prior to the assessment, the assessor demonstrates the walk at a comfortable walking pace. The outcome is the total time it takes to walk 20 meters. The assessor asks participants to complete two trials and computes the average time to complete the trials [61].

Two functional balance tests have been used: *Berg Balance Scale* and *Postural Sway* as measured by a Force Plate.

*The Berg Balance Scale* measures balance among older adults with balance impairment by assessing a participant’s performance during 14 functional tasks. The tasks include standing from a seated position, standing unsupported for 2 minutes, turning 360 degrees, and standing on one foot. The Berg Balance Scale has been evaluated in several reliability studies. Berg scores range from 0 to 56 and higher is better [62].

*Postural sway* is also used to determine balance by measuring the distance from center of pressure (CoP) defined as the vertical forces exerted by both feet on a force plate (Model BP5050, Bertec Corporation, Columbus, OH, USA). Similar to previous research [63], participants are asked to stand barefoot on the force plate with feet approximately at hip width and arms by their side. Participants are instructed to stand as stationary as possible for 30 seconds, repeated for 8 trials, alternating eyes open and eyes closed. The data are collected in both the anterior-posterior (CoPx) and medio-lateral (CoPy) axes at a sampling rate of 1000 Hz. Subjects’ postural stability is quantified as the mean standard deviation of CoPx and CoPy for eyes open and eyes closed trials.

#### Measures of muscle strength/power

Participants’ *Muscle Strength and Power* is measured using a leg press. Participants are seated on the bilateral leg press apparatus with knees flexed to 90 degrees and hips flexed to approximately 110 degrees (Leg Press A420, Keiser Corporation, Fresno, CA). Knee angle is measured using an electrogoniometer (ADInstruments, Colorado Springs, CO). Each participant is given the opportunity to familiarize themselves with the testing equipment through the use of a visual demonstration and practice at low resistances. Force, position, and velocity of each repetition are sampled at 400 Hz and saved to disk for offline analysis. Using software provided by the manufacturer, these data are then converted to force, position and velocity at the footplate (Software Release 7.8, Keiser Corporation, Fresno, CA). Leg extensor muscle strength are quantitatively assessed using the one-repetition maximum (1RM) technique and are defined as the maximum load that could be moved only once throughout the full range of motion (ROM) while maintaining proper form [64]. Subjects perform the concentric phase, maintain full extension, and perform the eccentric phase of each repetition over 2, 1, and 2 seconds, respectively. After measurement of the 1RM, assessment of leg press peak muscle power is made after a 5 minute rest period. Performance of this multiple attempt peak power test has been previously described and validated [64]. Briefly, each participant is instructed to complete a total of five repetitions each separated by 30 seconds as quickly as possible through their full ROM at both 70% and 40% of the 1RM. The highest measured power output is recorded as the leg press peak power.

#### Accelerometry

Accelerometry allows objective measurement of physical activity by the use of a motion sensor which records both the number and magnitude of vertical accelerations generated by human movement. This allows both volume and intensity of activity to be registered. In this study, accelerometry measurements are performed using the Actigraph Model 7164 (Manufacturing Technology Inc., FL, USA). The actigraph is worn superior to the iliac crest in a custom pouch, secured to the participant’s belt by a Velcro fastener. Participants are instructed to wear the actigraph during the baseline and follow-up visits for a consecutive 7-day period, excluding sleep and bathing time. Activity is recorded using 1-min epochs and participants also complete a log to record when the actigraph was worn.

#### Gait analysis

A gait analysis is a qualitative assessment to determine whether varus thrust is present. Varus thrust is defined as the visualized dynamic bowing-out of the knee laterally (i.e. the abrupt first appearance of varus) while the limb is in the weight-bearing (stance) phase of gait, followed by the return to a less varus alignment during the non- weight-bearing (swing) phase of gait. We will measure whether varus thrust is 1) definitely present 2) possibly present or 3) absent. These assessments will be made for both knees.

### Adherence

All participants are encouraged to maintain their usual physical activities, but to perform no new formalized exercise program. Participants randomized to Physical Therapy are asked not to perform Tai Chi and participants randomized to Tai Chi are asked not to do Physical Therapy for the study knee unless instructed to by a doctor. We track the number of missed sessions for each participant. Participants’ attendance is monitored during each in-person session (12 for Tai Chi and 6 weeks for Physical Therapy) by staff-completed attendance forms as well as class sign-in sheets for the Tai Chi intervention. Participants are also asked to maintain daily Tai Chi or Physical Therapy practice throughout the follow up period and are encouraged with phone calls from the research staff once a month until the end of the 52 weeks using standardized questionnaires,. Adherence is measured post-intervention by these calls during which research staff ask about the frequency and duration of the Tai Chi and Physical Therapy exercises as well as which exercises were done by those participants in the Physical Therapy intervention.

### Safety

Study participants are monitored weekly during the study intervention for the occurrence of adverse events defined as any undesirable experience. All adverse events are recorded on an adverse event case report form during study interventions and evaluated for relevance to the intervention and severity according to IRB mandated criteria by the study rheumatologist. These will be reported by category and are examined for trends that could indicate safety risk to the participants with particular emphasis on serious adverse events and any adverse events deemed related to the intervention. No systematic ascertainment of adverse events is undertaken after the intervention period (i.e., after 12 weeks) based on the principle that all would, by definition, be unrelated to the intervention. This plan has been approved by the ethics review board and the data and safety monitoring board.

### Data management

Study data are to be collected and managed using the REDCap electronic data capture system [65]. Participants who are not able to directly enter data into the Redcap system will be asked to fill out paper case report forms, which will be entered into REDCap by study staff. These case report forms will then be filed in the participants’ file and stored in the principal investigator or study coordinator’s office in locked filing cabinets. No participant identifiers are included in the study database. Data are exported from the database into statistical software for analysis. Additionally, REDCap has an audit trail that records every time a participant or staff member makes changes to any data entered on the website.

### Sample size

We proposed to enroll 180 participants, 90 in each arm. Power analyses are based on hypothesized changes in WOMAC pain, WOMAC function, chair-stand performance, and the SF-36 Physical Component Summary scores. We are using the outcomes of two RCTs that used attention controls to formulate expected effects. The first study by Baker et al. compared the effects of a 4–month, home-based Physical Therapy training program with attention control group among 46 older participants with knee OA. Researchers found that the Physical Therapy strength training group had an 80-point better mean change in the WOMAC pain score and a 270-point better mean change in the WOMAC physical function score [66]. The second study was our pilot trial of 40 participants with knee OA, comparing Tai Chi with attention control [18]. We found a 150-point better mean change in the WOMAC pain score and a 500-point better mean change for the WOMAC physical function scale in the Tai Chi group. Based on these data, we expect Tai Chi to be 70 points better than strength training on the WOMAC pain scale and 230 points better on the WOMAC physical function scale. A sample size of 76 per group has 99% power to detect these differences at 0.05 type I error level. For secondary outcomes such as the Chair Stand and quality of life (SF-36 Physical Component Score-PCS), a sample size of 76 per group has >90% power to detect the difference between these two groups. Although we did not have any attrition in our pilot study, we are conservatively assuming a 15% dropout rate. If the dropout rate exceeds our prediction, we will recalculate power effects to see if additional participants are needed. Also, if it is determined that more participants are needed to fill the sessions allowing for between instructor comparisons and comparable group dynamics, the sample size will be reevaluated. Furthermore, we plan to analyze the primary study outcome first for non-inferiority and then for superiority of the Tai Chi intervention. Using a non-inferiority limit of 20 in the WOMAC Pain score and a one-sided error rate of 5%, the proposed sample will provide greater than 95% power to detect non-inferiority if the planned intervention is only half as effective as expected (e.g. WOMAC Pain scores of 115 versus 80 with a standard deviation of 100). The study has 84% power to detect non-inferiority if the difference in WOMAC scores between treatments is as low as 20. If non-inferiority is found then the superiority of Tai Chi will be tested.

### Data and safety monitoring

We have established an NIH-approved independent data safety monitoring board (DSMB). The DSMB team is comprised of investigators with expertise in rheumatology clinical trials, statistical design, exercise, and adverse events. Members of the DSMB do not have any affiliation with Tufts Medical Center. The DSMB is responsible for monitoring the project, subject safety and adequacy of data quality. Specifically, DSMB responsibilities include the following:

1. Review the protocol, informed consent documents, and plans for data and safety monitoring.
2. Make recommendations on the study conduct, enrollment, sample size, and data collection.
3. Periodic evaluation of progress on the study, including assessments of data quality, recruitment and retention, and participant risk versus benefit.
4. The DSMB is charged with making the decision for termination or continuation of the trial based on the a priori rules and the data and informing the PI of their decision. In the event that the study is discontinued, the Tufts Medical Center and Tufts University Health Sciences Campus IRB and the participants are informed and intervention-related activities cease.
5. Consider external factors such as scientific or therapeutic developments that may impact the safety of the participants or the ethics of the trial.
6. Protect the safety and scientific progress of the trial.
7. Make recommendations to the PI regarding the appropriateness of continuation, termination, or other modifications to the trial based on observed beneficial or adverse effects of the investigational agent in accordance with clearly-defined data-driven algorithms.
8. Assure data integrity, confidentiality, and monitoring results.

The board meets at least once during the study and more frequently as needed. Decisions are made by a majority vote. Following each DSMB meeting, the Chair prepares a report to be submitted to the Tufts Medical Center and Tufts Health Sciences Campus IRB by the PI. All materials, discussions, and proceedings of the DSMB are highly confidential. The DSMB chair receives reports of all serious events throughout the conduct of the study. We provide to the DSMB a number of reports including serious adverse events or death within 24 hours of knowledge of event occurrence, annual reports of all adverse events as well as routine progress reports prior to each DSMB meeting.

### Analysis

The primary outcome is the measurement of change in knee pain between baseline and 12 weeks. The primary analysis of this outcome will be an intent-to-treat analysis in which all participants are analyzed in the group to which they are randomized with no adjustment for covariates and with missing 12-week outcomes imputed as no change. Secondary analyses will adjust for covariates whose baseline distributions differ by treatment group and will explore potential interactions of these covariates with treatment. Such baseline characteristics could include gender, baseline pain severity, opiate use, disease duration, and number of chronic conditions. Because we are interested in potential effects by instructor as well as differences by the two Physical Therapy sites, we will also form contrasts of these strata within treatment groups to explore differences.

Change in knee pain from baseline at 24 weeks and 52 weeks are secondary outcomes as are all other outcomes (Table 1). These will be analyzed in the same way as the primary outcome. In addition, we will analyze the four time points in one longitudinal analysis in order to detect potential time trends and interactions of treatment effect with time. These longitudinal analyses will use mixed models with random effects for patient and fixed effects for time and will explore autoregressive correlation structures to explain residual error.

These will be analyzed both as individual time points and in longitudinal analyses. Missing values will be imputed by multiple imputations assuming data are missing at random. Because some individuals may drop out early because of poor outcomes, we will also investigate missing not at random models based on informative dropout models. As part of these sensitivity analyses for missing data, we will explore Bayesian models that can incorporate prior assumptions about the non-ignorable missing data mechanisms [67].

### Cost-effectiveness analysis

We will also perform an economic analysis to evaluate the cost-effectiveness of Tai Chi versus Physical Therapy for patients with knee OA. Based on the health care utilization during the trial using the full Health Assessment Questionnaire, we will apply standard microcosting methods from the US Panel on Cost-effectiveness in Health and Medicine [68]. Cost-effectiveness analyses have become a well-accepted component of clinical trials with the capture of health resource use and health state utilities directly from study subjects [69]. For each individual, we will focus on the following major and readily captured medical and productivity measures: Physical Therapy and Tai Chi session costs, medications, emergency room visits, outpatient visits, hospitalizations and productivity (full-time, part-time work). Cost-effectiveness analysis will assess the trial primary and secondary outcomes based on the duration of the trial itself first and then an extension of projected outcomes beyond the observed trial follow-up, so these analyses will examine the direct and indirect costs and the reduction in pain, improvement in physical or psychosocial function. If outcomes with Tai Chi are improved and costs reduced versus Physical Therapy, then Tai Chi would be cost-saving, but if Tai Chi is more expensive, then incremental cost-effectiveness ratios will be determined for each outcome. Because the proposed trial is limited in the duration of observation yet induced benefits or costs may extend beyond the time horizon of the study, we will project the future implications of the observed trial outcomes on long-term health and costs using computer simulation models. To estimate the likely outcomes resulting from the alternative approaches, we will apply decision analysis, a prescriptive, normative approach to decision-making in the face of uncertainty. For these projected costs and outcomes, we will perform the following: (1) a systematic literature search to identify existing data to estimate model parameters (costs, natural history of knee osteoarthritis, disability related to osteoarthritis, knee replacement) and previously published economic models, (2) data synthesis to estimate means and ranges of uncertainty, and (3) Markov and Monte Carlo simulation model development based on the primary trial and literature data. Thus, the outcome of the cost-effectiveness analysis becomes incremental cost-utility analysis and is expressed as the cost per additional discounted quality-adjusted life year gained permitting comparison with the cost-effectiveness of other medical interventions. Medical treatment or screening interventions are typically considered to be "cost-effective" in the United States if they fall below $50,000 to $100,000 per discounted quality-adjusted life year gained, a range in which many well-accepted medical strategies fall. Because uncertainty surrounds all data estimates, extensive sensitivity analyses are performed varying each variable or groups of variables over their plausible ranges to determine their impact on the cost-effectiveness of the alternative strategies.

## Discussion

Finding new and cost-effective treatments to maintain function and quality of life in people with knee OA is a national priority. In this project, we are conducting the first randomized comparative effectiveness and cost-effectiveness trial. When completed, the study will be the largest mind-body therapy study which compares to a standardized physical therapy regimen on symptomatic knee OA population with long-term follow up. Our robust and well-designed randomized comparative-effectiveness trial will fill a critical knowledge gap in this field, and results of this study are likely to have significant public health implications.

In addition, testing other secondary outcomes based on the proposed larger sample size will allow us to address unanswered questions remaining from our previous studies, for instance, observed benefit dependent on instructors themselves and whether such observed benefit can be generalized to other populations. Thus, successful completion of the proposed study will contribute to the evidence base of whether Tai Chi instead of physical therapy can be used as a simple, inexpensive, effective, durable treatment for a major disabling disease simultaneously saving economic costs in the healthcare system. The study will be completed by September 2014.

## Electronic supplementary material

Additional file 1: **Subject Pre-Screening Interview.** (DOC 76 KB)

Additional file 2: **Instructions for Tai Chi Instructors.** (DOCX 32 KB)

Additional file 3: **Physical Therapy: Knee Evaluation/Re-evaluation.** (DOC 270 KB)

Additional file 4: **Physical Therapy for Knee Osteoarthritis Program.** (DOC 42 KB)
