# Supplementary material for: Assessing the comparative effectiveness of Tai Chi versus physical therapy for knee osteoarthritis: design and rationale for a randomized trial
Source: BMC Complement Altern Med. 2014 Sep 8;14:333. doi: 10.1186/1472-6882-14-333 (PMC4171546; doi:10.1186/1472-6882-14-333)
Supplement: Supplementary file 1 — Additional file 1: Subject Pre-Screening Interview. (DOC 76 KB) [file 12906_2014_1908_MOESM1_ESM.doc]

**Additional file 1: SUBJECT PRE-SCREENING INTERVIEW**

Date: __ __/__ __/__ __ (mm/dd/yy) **Eligibility: YES**  **NO**  **MAYBE**

Name:____________________________________________________ DOB: __ __/__ __/__ __

(First) (M.I.) (Last) (mm/dd/yy)

Address:__________________________________________________________ Gender: M / F (circle)

(Street) (City) (State) (Zip)

Phone: (d)_________________ (e)_________________ (cell)___________________

Hispanic: Yes / No (circle) Language spoken:_______________ Race:________________

**Medical Hx:**
(1) Have you ever been diagnosed by a physician as having OA of the knee?
YES  NO  Don’t Know  | Right Knee  Left Knee
Who made dx of Knee OA?____________________________________________________

Date of dx: __ __/__ __ (mm/yyyy) Don’t Know

(2) During the last month, did you have any knee pain or discomfort when walking 2 – 3 blocks (1/4 mile)?

YES  NO  Don’t Know

(3) Have you ever had a knee x-ray demonstrating evidence of knee OA?

YES  NO  Don’t Know

If **YES**, have you had an x-ray in the past two years? YES  NO  Don’t Know

Was your X-ray at Tufts Medical Center? YES  NO  Don’t Know

If **YES**: What is your medical record number? _______________________________

If **YES**: Do we have your permission to view your x-ray prior to your baseline evaluation, to determine likely eligibility for the study? YES  NO

(4) Do you take medications regularly for your knee pain?
 YES  NO  Don’t Know

(5) Have you had knee surgery in the last 3 months?

YES  NO  Don’t Know

If **YES**, which knee? Right Knee  Left Knee

If **YES**, what type? Arthroscopy  Osteotomy  Knee Replacement

Ligament Repair Surgery  Meniscectomy

(6) Have you **EVER** had knee replacement surgery? YES  NO  Don’t Know

If **YES**, which knee? Right Knee  Left Knee

(7) Are you considering having a knee replacement surgery in the next year? If **YES**, which knee?

YES  NO  Right  Left

(8) Are you planning to relocate in the next 12 months?
 YES  NO

(9) Do you have any medical conditions that limit your ability to participate in exercise safely?

YES  NO

If **YES**, what? ____________________________________________________________

(10) Do you use any assistive devices like a cane, crutches, or a knee brace?

YES  NO  Don’t Know

If **YES**, do you use the device ALL THE TIME, or are you able to walk without it?

All the time  Able to walk without device

(11) Have you had any knee injections in the past 6 months? YES  NO  Don’t Know

If **YES**, which knee? Right Knee  Left Knee

If **YES**, what type of injection? Synvisc  Hyalgan  Steroid

Other  Don’t Know

(12) Do you plan on having a knee injection? YES  NO  Don’t Know
If **YES**, when? Date: _______________

If **YES**, which knee? Right Knee  Left Knee

If **YES**, what type of injection? Synvisc  Hyalgan  Steroid

Other  Don’t’ Know

**Tai Chi/Physical Therapy Hx:**

Prior Experience with Tai Chi in the past 1 year……………… .YES  NO  Don’t Know

Prior Experience with other similar types
of CAM in the past 1 year such as Qi gong and yoga ………. YES  NO  Don’t Know

Prior Experience with Physical Therapy for the knee in the

past 1 year …. ……………………………………………… .YES  NO  Don’t Know

Involvement in any other studies in the last 30 days………… YES  NO  Don’t Know

Availability twice a week in the afternoon for 1 hour each…. YES  NO  Don’t Know

**Logistical:**

How did you hear about our study? ______________________

Have you ever been a patient at Tufts Medical Center? YES  NO  Don’t Know

Participated in previous Tai Chi trial at TMC? YES  NO

Entered into Pre-screening log:  Date: ________________
