# Supplementary material for: Assessing the comparative effectiveness of Tai Chi versus physical therapy for knee osteoarthritis: design and rationale for a randomized trial
Source: BMC Complement Altern Med. 2014 Sep 8;14:333. doi: 10.1186/1472-6882-14-333 (PMC4171546; doi:10.1186/1472-6882-14-333)
Supplement: Supplementary file 3 — Additional file 3: Physical Therapy: Knee Evaluation/Re-evaluation. (DOC 270 KB) [file 12906_2014_1908_MOESM3_ESM.doc]

**Additional file 3:** Physical Therapy: Knee Evaluation/Re-evaluation

| Date: (MM/DD/YYYY) | | | |
| --- | --- | --- | --- |
| Time: | | | MD: |
| Age: | Sex: M F | Diagnosis: | |
| Precautions: | | | |
| Occupation: | | | |

1. Involved Knee: Right _1_  <INPUT TYPE=\ Left 2 <INPUT TYPE=\ Bilateral 3 <INPUT TYPE=\

| **A. History** |
| --- |

2. Xrays No _1_  <INPUT TYPE=\ Yes 2 <INPUT TYPE=\ Comment: _____________________________

3. MRIs No _1_  <INPUT TYPE=\ Yes 2 <INPUT TYPE=\ Comment: _____________________________

4. Past Medical and Social History:

____________________________________________________________________________

_____________________________________________________________________________

5. Chief Complaint: ______________________________________________________________________________

6. Patient’s Expectations for Therapy:

_____________________________________________________________________________

1. Has the subject taken any medications for knee pain today? No 1 <INPUT TYPE=\ Yes 2 <INPUT TYPE=\

| **B. INDEX KNEE PAIN -** Past 24 hours |
| --- |

***Visual Analog Scale****: Rate 0-10 (with 10= worst pain)*

2. Highest pain with activity: _________ 2a. List activity that causes pain: _______________

3. Lowest pain with rest: _________

4. Location of knee pain (Check all that apply):

**Right** a  **Left b**

anterior_1_  <INPUT TYPE=\ posterior 2 <INPUT TYPE=\ jt line 3 <INPUT TYPE=\ anterior 1 <INPUT TYPE=\ posterior 2
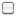
 jt line 3 <INPUT TYPE=\

medial_4_<INPUT TYPE=\ sup5<INPUT TYPE=\ lat_6_<INPUT TYPE=\ Infer to pat_7_
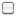
 medial4 <INPUT TYPE=\ sup5
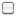
 lat_6_<INPUT TYPE=\ Infer to pat7
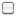


Criterion for recurvatum is knee ROM hyperextension greater than 0°

| **C. KNEE ALIGNMENT**  *Posture: Standing, No Shoes, Feet Shoulder Width Apart* |
| --- |

1. Right Genu Recurvatum No 1 <INPUT TYPE=\ Yes 2 <INPUT TYPE=\

2. Left Genu Recurvatum No 1 <INPUT TYPE=\ Yes 2 <INPUT TYPE=\

*Criterion for valgus is > 3.5 inches between malleoli when knees are together*

3. Right Genu Valgus No 1 <INPUT TYPE=\ Yes 2 <INPUT TYPE=\

4. Left Genu Valgus No 1 <INPUT TYPE=\ Yes 2 <INPUT TYPE=\

Criterion for varus is > 3 fingers apart at the knee with ankles together

5. Right Genu Varus No 1 <INPUT TYPE=\ Yes 2 <INPUT TYPE=\

6. Left Genu Varus No 1 <INPUT TYPE=\ Yes 2 <INPUT TYPE=\

1. Right foot Normal 1 <INPUT TYPE=\ Planus 2 <INPUT TYPE=\ Cavus 3<INPUT TYPE=\

| **D. FOOT ALIGNMENT**  *Posture: Standing, No Shoes, Feet Shoulder Width Apart* |
| --- |

2. Left foot Normal 1 <INPUT TYPE=\ Planus 2 <INPUT TYPE=\ Cavus 3<INPUT TYPE=\

1. Right Knee Edema None _1_  <INPUT TYPE=\ 1+ 2 <INPUT TYPE=\ 2+ _3_  <INPUT TYPE=\ 3+ 4 <INPUT TYPE=\

| **E. EDEMA, SKIN INTEGRITY, TENDERNESS, SENSATION** |
| --- |

2. Left Knee Edema None _1_  <INPUT TYPE=\ 1+ 2 <INPUT TYPE=\ 2+ _3_  <INPUT TYPE=\ 3+ 4 <INPUT TYPE=\

3. Right Knee Scar No _1_  <INPUT TYPE=\ Yes 2 <INPUT TYPE=\

4. Left Knee Scar No _1_  <INPUT TYPE=\ Yes 2 <INPUT TYPE=\

5. Right knee Palpation/Tenderness Negative _1_  <INPUT TYPE=\ Positive 2 <INPUT TYPE=\ Location: ___________

6. Left Knee Palpation/Tenderness Negative _1_  <INPUT TYPE=\ Positive 2 <INPUT TYPE=\ Location: ___________

7. Right Knee Sensation WNL_1_  <INPUT TYPE=\ Decreased 2 <INPUT TYPE=\ Increased 3 <INPUT TYPE=\ Absent4
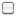


8. Left Knee Sensation WNL_1_  <INPUT TYPE=\ Decreased 2 <INPUT TYPE=\ Increased 3 <INPUT TYPE=\ Absent4
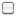


1. Right Leg Length Trial 1 __________cm Trial 2 ________cm Avg ________cm

| **F. LEG LENGTH DISCREPANCY**  *Supine below ASIS to below malleoli* |
| --- |

2. Left Leg Length Trial 1 _________cm Trial 2 ________ cm Avg ________cm

| **G. CONTOUR**  *Assessed Supine- mark patella and leave tape in place when flex knee* |
| --- |

1. Right Knee mid patella ext___________ cm full flexion___________ cm

2. Left Knee mid patella ext___________ cm full flexion___________ cm

1. Right Knee deg of flexion contracture ______ full flexion___________ hypertext ______

| **H. KNEE PASSIVE RANGE OF MOTION**  *Supine*  *If full range of motion write in 0 for contracture* |
| --- |

2. Left Knee deg of flexion contracture ______ full flexion___________ hypertext ______

1. Vastus Medialis Oblique Contraction No 1 <INPUT TYPE=\ Yes 2 <INPUT TYPE=\ Weak 3
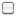


| **I. MUSCLE LENGTH ABNORMALITIES** |
| --- |

*AKE test position - Supine- Active* *(Positive > 20 degrees knee flexion measured by goniometer)*

2. Right 90-90 Hamstring AKE Muscle length WNL 1 <INPUT TYPE=\ Positive 2 <INPUT TYPE=\

3. Left 90-90 Hamstring AKE Muscle length WNL 1 <INPUT TYPE=\ Positive 2 <INPUT TYPE=\

*Gastrocnemius test position - Supine-Passive, knee ext, ankle df (+ if < 10 deg df measured by goniometer)*

4. Right Gastrocnemius WNL 1 <INPUT TYPE=\ Positive 2 <INPUT TYPE=\

5. Left Gastrocnemius WNL 1 <INPUT TYPE=\ Positive 2 <INPUT TYPE=\

*Ely test position - Prone-Passive (Positive: same side hip flexes before full knee flexion)*

6. Right Ely test WNL 1 <INPUT TYPE=\ Positive 2 <INPUT TYPE=\

7. Left Ely test WNL 1 <INPUT TYPE=\ Positive 2 <INPUT TYPE=\

*Ober Test Position - Patient on side, unaffected leg on bottom and bent; affected leg on top and straight. Stabilizing hand on patient's upper iliac crest, lift straight upper leg, extends at hip and slowly lower it behind bottom leg, allowing it to adduct below and behind the exam table. Positive: If patient can't adduct the leg past exam table.*

8. Right Ober test WNL 1 <INPUT TYPE=\ Positive 2 <INPUT TYPE=\

9. Left Ober test WNL 1 <INPUT TYPE=\ Positive 2 <INPUT TYPE=\

**Right Left**

| **J. Special Tests** |
| --- |

1. Lachmans Pos1<INPUT TYPE=\ Neg 2 <INPUT TYPE=\ NA4 <INPUT TYPE=\ Pos1<INPUT TYPE=\ Neg 2 <INPUT TYPE=\ NA4 <INPUT TYPE=\

2. Posterior sag Pos1<INPUT TYPE=\ Neg 2 <INPUT TYPE=\ NA4 <INPUT TYPE=\ Pos 1<INPUT TYPE=\ Neg 2 <INPUT TYPE=\ NA4 <INPUT TYPE=\

3. MCL (at 0 deg) Pos1<INPUT TYPE=\ Neg 2 <INPUT TYPE=\ NA4 <INPUT TYPE=\ Pos 1<INPUT TYPE=\ Neg 2 <INPUT TYPE=\ NA4 <INPUT TYPE=\

3b. MCL (at 30 deg) Pos1<INPUT TYPE=\ Neg 2 <INPUT TYPE=\ NA4 <INPUT TYPE=\ Pos 1<INPUT TYPE=\ Neg 2 <INPUT TYPE=\ NA4 <INPUT TYPE=\

4. LCL (at 0 deg) Pos1<INPUT TYPE=\ Neg 2 <INPUT TYPE=\ NA4 <INPUT TYPE=\ Pos 1<INPUT TYPE=\ Neg 2 <INPUT TYPE=\ NA4 <INPUT TYPE=\

4b.LCL (at 30 deg) Pos1<INPUT TYPE=\ Neg 2 <INPUT TYPE=\ NA4 <INPUT TYPE=\ Pos 1<INPUT TYPE=\ Neg 2 <INPUT TYPE=\ NA4 <INPUT TYPE=\

6. McMurry’s Pos1<INPUT TYPE=\ Neg 2 <INPUT TYPE=\ NA4 <INPUT TYPE=\ Pos 1<INPUT TYPE=\ Neg 2 <INPUT TYPE=\ NA4 <INPUT TYPE=\

7. Apley’s Compres Pos1<INPUT TYPE=\ Neg 2 <INPUT TYPE=\ NA4 <INPUT TYPE=\ Pos 1<INPUT TYPE=\ Neg 2 <INPUT TYPE=\ NA4 <INPUT TYPE=\

8. Apley’s Distract Pos1<INPUT TYPE=\ Neg 2 <INPUT TYPE=\ NA4 <INPUT TYPE=\ Pos 1<INPUT TYPE=\ Neg 2 <INPUT TYPE=\ NA4 <INPUT TYPE=\<INPUT TYPE=\

1. Able to perform Waldron sign? Yes 1 <INPUT TYPE=\ Yes, with support 2
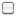
 NO 3 <INPUT TYPE=\ NA 4 <INPUT TYPE=\

| **K Patella Dysfunction** Standing Positive: pain and crepitus with bilateral squat |
| --- |

2. Right Waldron Sign WNL 1 <INPUT TYPE=\ Positive 2 <INPUT TYPE=\ NA 4 <INPUT TYPE=\

3. Left Waldron Sign WNL 1 <INPUT TYPE=\ Positive 2 <INPUT TYPE=\ NA 4 <INPUT TYPE=\

4. Right Grind Test WNL 1 <INPUT TYPE=\ Positive 2 <INPUT TYPE=\ NA 4 <INPUT TYPE=\

5. Left Grind Test WNL 1 <INPUT TYPE=\ Positive 2 <INPUT TYPE=\ NA 4 <INPUT TYPE=\

*Right Left*

| **L. MUSCLE STRENGTH** traditional 0-5 scale Code- WNL = 5 WFL = 4 |
| --- |

1. Dorsiflexion ______ ________

2. Plantarflexion ______ ________

3. Inversion ______ ________

4. Eversion ______ ________

5. Knee flexion ______ ________

6. Knee extension ______ ________

7. Hip flex/ext ______/_____ ______/_____

8. Hip abduction/add ______/_______ ______/____

9. Hip IR/ER ______/______ ______/______

Upper Extremity/Trunk Strength: _____________________________________________________________________________

1. Pain with descending stairs? NO 1 <INPUT TYPE=\ YES 2 <INPUT TYPE=\

| **M. Functional Activities-** asked by PT |
| --- |

2. Pain with ascending stairs? NO 1 <INPUT TYPE=\ YES 2 <INPUT TYPE=\

3. Uses banister when ascending stairs? NO 1 <INPUT TYPE=\ YES 2 <INPUT TYPE=\

4. Uses banister when descending stairs? NO 1 <INPUT TYPE=\ YES 2 <INPUT TYPE=\

5. Observed Gait: Normal 1 <INPUT TYPE=\ Antalgic gait 2 <INPUT TYPE=\ Ambulates with device 3 <INPUT TYPE=\

Ambulates with brace on right 3<INPUT TYPE=\
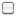
 Ambulates with brace on left 3<INPUT TYPE=\
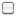


6. If ambulates with device, describe device (check all that apply):

<INPUT TYPE=\ Cane or one crutch1
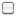
 Crutches 2 <INPUT TYPE=\ <INPUT TYPE=\ Walker 3
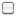


7. Weightbearing Status Right: Full 1 <INPUT TYPE=\ Partial 2 <INPUT TYPE=\ TD3 <INPUT TYPE=\

8. Weightbearing Status Left: Full 1 <INPUT TYPE=\ Partial 2 <INPUT TYPE=\ TD3 <INPUT TYPE=\

Comments: _____________________________________________________________________________

_____________________________________________________________________________

Patient Education:

_____________________________________________________________________________
