# Supplementary material for: Assessing the comparative effectiveness of Tai Chi versus physical therapy for knee osteoarthritis: design and rationale for a randomized trial
Source: BMC Complement Altern Med. 2014 Sep 8;14:333. doi: 10.1186/1472-6882-14-333 (PMC4171546; doi:10.1186/1472-6882-14-333)
Supplement: Supplementary file 4 — Additional file 4: Physical Therapy for Knee Osteoarthritis Program. (DOC 42 KB) [file 12906_2014_1908_MOESM4_ESM.doc]

**Additional file 4**

**Physical Therapy For Knee Osteoarthritis Program***

| **Exercise Description**  **Calf Stretching:** Subject stands in front of wall with hands supporting body against the wall. For the limb being stretched, the hip is extended, the knee is extended and the foot is placed flat on the floor. The contralateral limb rests on the floor for stability with the hip and knee comfortably flexed and the foot resting comfortably on the floor. The patient slowly leans forward toward the wall, keeping the foot flat and maintained in slight supination, and keeping the knee extended, until a stretch discomfort is felt by the subject in the calf muscles.  **Hamstring Stretching:** The therapist stabilizes the contralateral limb on the plinth and moves the stretching limb in a straight leg raise position, flexing the hip until a stretch discomfort is felt by the subject in the hamstrings while keeping the knee in full extension. The range can be increased during the 30 second period if the patient reports that the stretch discomfort has decreased.  **Prone Quadriceps Stretching**: The subject lies on the treatment table in prone. The therapist stabilizes the contralateral limb on the plinth. The knee of the stretching limb is placed in 90 degrees of flexion, then the therapist extends the hip until a stretch discomfort is felt by the subject in the quadriceps  **Long-Sitting Knee Flexion and Extension:** The subject is positioned in long-sitting on the treatment table. The therapist instructs the subject to flex the knee as far as possible by sliding the foot along the treatment table toward the pelvis. The subject holds the flexed position for 3–5 seconds. A belt, towel, or a strap may be used by the subject to assist with bending the knee. The therapist then instructs the subject to extend the knee by sliding the foot along the treatment table toward the end of the table. The subject holds the fully extended position for 3–5 seconds.  **Quadriceps setting:** The subject is positioned in long sitting with the knee extended. Therapist instructs the subject to isometrically contract the quadriceps muscles bilaterally as vigorously as possible without reproducing pain. The subject is instructed to hold the contraction for 3–5 seconds.  **Supine straight leg raises:** The subject is positioned in supine on the treatment table. The contralateral knee is flexed so that the foot is resting comfortably in a foot flat position on the table. The therapist instructs the subject to raise the exercise limb with the knee maintained in full extension to the height of the contralateral flexed knee position, then lower the limb back to the table  **Seated knee extension isometrics:** The subject is seated on a leg extension exercise device with the knee positioned in a comfortable flexed position between 90° and 60° of flexion. The subject is instructed to push against the force pad of the extension device as vigorously as possible without reproducing pain symptoms. The subject is instructed to hold the contraction for 3–5 seconds.  **Prone straight leg raises:** The subject is positioned in prone on the treatment table. The therapist instructs the subject to raise the exercise limb with the knee maintained in full extension as high as possible, then lower the limb back to the table.  **Standing Calf Raises:** The subject is positioned in standing with both feet flat on the floor. The subject is instructed to raise up on the toes as high as possible, holding for 1–2 seconds then return to the foot flat position. | **Exercise Dosage/Progression**  2 repetitions, each of 30 seconds duration. Performed on both limbs. Range can be increased during 30 second period if subject reports stretch discomfort has decreased.  2 repetitions, each of 30 seconds duration. Performed on both limbs. Range can be increased during 30 second period if subject reports stretch discomfort has decreased.  2 repetitions, each of 30 seconds duration. Performed on both limbs. Range can be increased during 30 second period if subject reports stretch discomfort has decreased. Repetitions are progressed from a minimum of 10 to a maximum of 30 reps. Exercise is repeated on the opposite limb. Exercise is progressed from 10 contractions to 30 contractions as tolerated.  Exercise is progressed from 10 to 30 reps. When subject can do 30 reps without added weight, a 1 pound cuff weight is added. Resistance is progressed by adding 1 pound when the subject can do 30 reps at the current resistance. Exercise is performed on each limb.  Exercise is progressed from 10 contractions to 30 contractions as soon as possible (by the 3^rd^ treatment visit). Exercise is performed on each limb.  Repetitions are progressed from a minimum of 10 to a maximum of 30 reps. |
| --- | --- |

* Brief examples of the flexibility and straight plane strengthening exercises that modified from Scopaz et al. Arthritis & Rheumatism 2009; 61(7):951-957, doi: [10.1002/art.24650](http://dx.doi.org/10.1002%2Fart.24650). Note the home program is tailored with functional activities, balance and proprioceptive exercises.
